# Transmission dynamics of Zika virus with multiple infection routes and a case study in Brazil

**DOI:** 10.1038/s41598-024-58025-7

**Published:** 2024-03-28

**Authors:** Liying Wang, Qiaojuan Jia, Guanghu Zhu, Guanlin Ou, Tian Tang

**Affiliations:** 1 Key Laboratory of Cognitive Radio and Information Processing, Ministry of Education (Guilin University of Electronic Technology), Guilin, 541004 China; 2 School of Mathematics and Computing Science, Guilin University of Electronic Technology, Guilin, 541004 China; 3 School of Information and Communication, Guilin University of Electronic Technology, Guilin, 541004 China

**Keywords:** Zika virus, Transmission dynamics, Infection risk, Basic reproduction number, Infectious diseases, Risk factors, Infection, Applied mathematics

## Abstract

The Zika virus (ZIKV) is a serious global public health crisis. A major control challenge is its multiple transmission modes. This paper aims to simulate the transmission patterns of ZIKV using a dynamic process-based epidemiological model written in ordinary differential equations, which incorporates the human-to-mosquito infection by bites and sewage, mosquito-to-human infection by bites, and human-to-human infection by sex. Mathematical analyses are carried out to calculate the basic reproduction number and backward bifurcation, and prove the existence and stability of the equilibria. The model is validated with infection data by applying it to the 2015–2016 ZIKV epidemic in Brazil. The results indicate that the reproduction number is estimated to be 2.13, in which the contributions by mosquito bite, sex and sewage account for 85.7%, 3.5% and 10.8%, respectively. This number and the morbidity rate are most sensitive to parameters related to mosquito ecology, rather than asymptomatic or human-to-human transmission. Multiple transmission routes and suitable temperature exacerbate ZIKV infection in Brazil, and the vast majority of human infection cases were prevented by the intervention implemented. These findings may provide new insights to improve the risk assessment of ZIKV infection.

## Introduction

Zika virus (ZIKV) is a Flavivirus closely related to dengue, which was first discovered in 1947 in Uganda among a certain Rhesus macaque population^1^. However, few human infections were reported until 2015, when ZIKV infection unexpectedly struck the Americas and spread to other countries over the next 2 years^2,3^. The WHO has recorded that a total of 87 countries and territories have reported evidence of autochthonous ZIKV infection, with more than 1.4 million suspected and confirmed Zika cases^4^. ZIKV is a serious public health problem, which has the following concerns: (1) large number of human infections with ZIKV (0.4–1.3 million cases in Brazil alone)^3^; (2) serious consequence of infection, in which ZIKV infection during pregnancy can cause microcephaly and other congenital anomalies in developing fetuses and newborns (nearly 6000 suspected cases of microcephaly among newborns might be linked to ZIKV infections in Brazil during 2015–2016^5,6^); (3) about three quarters of cases of ZIKV infection are asymptomatic, who can transmate the disease but not easy to be identified^7,8^; (4) multiple transmission routes, where human can be infected through mosquitoes or humans, adult mosquitoes can be infected through humans, and larvae mosquitoes can be infected through the virus sewage^9–15^. Understanding such complex transmission patterns can provide scientific evidence to guide disease control.

Existing studies mainly use epidemiological investigation, statistical approach and mathematical models to tackle the above-mentioned issues^9–19^. By addressing the impacts of multiple transmission routes on ZIKV infection, it is found that (1) sexual transmission increases the risk of infection and epidemic size, and prolongs the outbreak^9^; (2) prevention and control efforts against ZIKV should target both the mosquito-borne and sexual transmission routes^9^; and (3) scenario exploration indicates that personal protection could be more effective than mosquito-reduction intervention^10^, and releasing male Wolbachia mosquitoes may be a good choice of disease control^12^. Furthermore, experimental studies have shown that urine from infected humans could be a natural ZIKV source, where Aedes mosquitoes are permissive to ZIKV infection when breeding in urine or sewage containing low concentrations of ZIKV^13,14^. However, there is a lack of mechanistic frameworks for modeling different routes of ZIKV transmission, and fewer evaluations of these routes on infections^9,11,12,15^.

To fill the knowledge gap, this study proposes a mechanistic framework for modeling ZIKV transmission patterns by addressing the following questions: How to account for the vector–virus–host interactions and the multiple routes of infection and thus to simulate the diffusion process? How to calculate the dynamics of transmission and assess the risk of infection, and thus suggest how to intervene? How to characterize the role of the dominant factors in activating or inhibiting the disease evolution? Answering these questions may provide new insights to improve ZIKV risk assessment and guide disease control. Mathematical model and data fitting method are employed to tackle these challenges. Specifically, based on the classical Ross–Macdonald theory and compartmental principle of mosquito-borne disease^20^, a new ZIKV transmission model is established by ordinary differential equations (ODEs), which systematically combines the dynamics of virus evolution, multiple infection routes, mosquito ecology and human behavior. Since temperature plays a vital role in ZIKV infection, its effects is included by modulating the transmission factors of virus and mosquitoes (oviposition rate, aquatic transition rate, hatching rate, mosquito bite rate and infection rate). Mathematical analysis are used to explore the transmission dynamics, including the expression of the basic reproduction number, the existence of bifurcation, and the stability of the equilibria. By using Markov Chain Monte Carlo (MCMC) methods to fit the time series of the reporting cases, the model is further validated to analyze the ZIKV outbreak in Brazil during 2015–2016. Numerical simulations and sensitivity analysis are performed to illustrate the detailed transmission patterns in Brazil.

This paper is organized as follows. The model is presented in “Modeling framework” and analyzed mathematically in “Mathematical analysis”. Main results are presented in “Model application”, including simulation ZIKV transmission in Brazil, the impact of transmission routes on the epidemic and the effectiveness of epidemic prevention and control. A brief discussion is presented in the last section.

## Modeling framework

A mechanistic framework for simulating ZIKV transmission patterns is established in this section. Inspired by recently-developed mathematical models^9,16–19^, and based on epidemiological feature of ZIKV, a new dynamic system is proposed, which takes into account human-to-human (sexual transmission) and human-to-mosquito (bite transmission and sewage transmission) interactions by using compartmental and deterministic principle. The total numbers of human, larval and adult mosquitoes are represented by ${{N}_{h}}$, ${{M}_{v}}$ and ${{N}_{v}}$. Given the stability of human demographics and the extremely low rate of ZIKV-related mortality, ${{N}_{h}}$ is assumed to be a constant.

Based on the characteristics of ZIKV infection, it is further assumed that: (1) the humans are divided into five categories: susceptible ${{S}_{h}}$, latent ${{E}_{h}}$, symptomatic infected ${{I}_{s}}$, asymptomatic infected ${{I}_{a}}$ and recovered ${{R}_{h}}$; (2) the larval stage can be divided into two categories: uninfected ${{A}_{v}}$ (including eggs, larvae and pupae) and infected ${{J}_{v}}$; (3) the adult female mosquitoes are divided into three categories: susceptible ${{S}_{v}}$, latent ${{E}_{v}}$ and infected ${{I}_{v}}$.

It is known that mosquito oviposition is linked to mature females and the ability of mosquitoes to develop oviposition habitats. If there are too many eggs in the oviposition habitat or too few nutrients and water resources, the females will lay fewer eggs or choose another site^21,22^. In addition, larvae and pupae need water or nutrients complete their development^21^. This biological phenomenon can be expressed by a Logistic model which explicitly incorporates the idea of limited carrying capacity resources^21,22^. Hence, the per capita oviposition rate is given by $\theta ( 1-({A}_{v}+{J}_{v})/K ){N}_{v}$.

**Figure 1:**
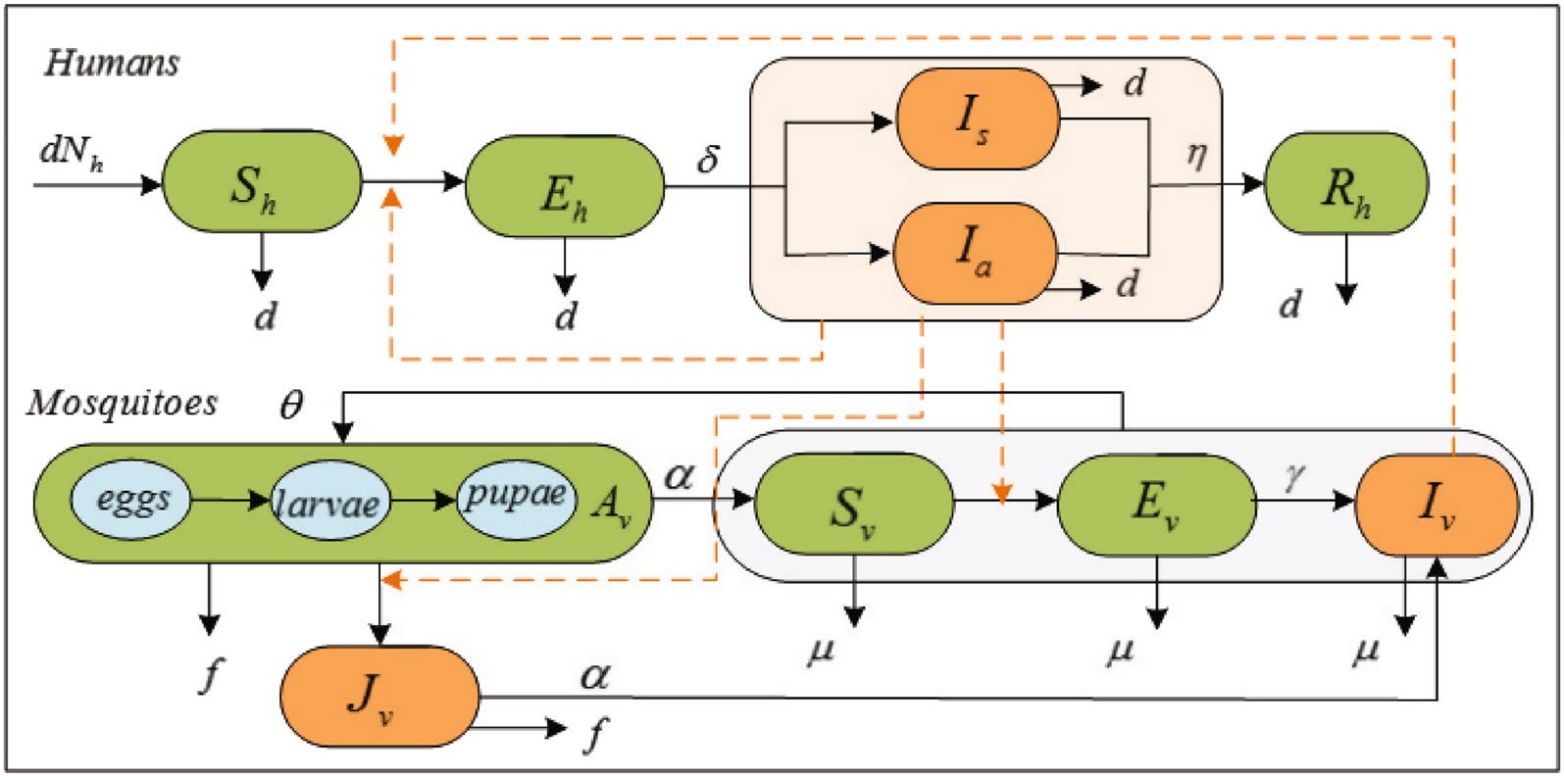
Flow diagram of the ZIKV transmission among humans and mosquitoes. The black solid lines indicate the progress of infection and ecology. The yellow lines indicated the infection pathes.

When ZIKV invades an area, humans and mosquitoes there could be infected with certain probability. It is governed by the following rules. A susceptible human may be infected with ZIKV from the bite of infectious mosquitoes at rate *b* or through human contact with infected people at rate $\beta$. Infected humans undergo an incubation period ${1}/{\delta }$. After that they could be symptomatic $\phi$ and asymptomatic $1-\phi$, and then the former follows by an infectious period of mean duration ${1}/{\eta }$ until recovery. The larvae may be directly infected by the virus sewage at rate *p* and grow up to become infectious adult mosquitoes at rate $\alpha$. Susceptible mosquitoes become infected at rate *a* by biting infectious humans, and the infected mosquito experience an extrinsic incubation period ${1}/{\gamma }$, that is followed by an infectious state from which they do not recover. The mean larvae lifespan and adult lifespan are 1/*f* and $1/\mu$, respectively. Flow chat is shown in Fig. 1.

Accordingly, the following ODEs are used to simulate the transmission dynamics of ZIKV:

1 $$\begin{aligned} \left\{ \begin{array}{ll} \displaystyle \frac{d{{S}_{h}}}{dt}=d{{N}_{h}}-bc\frac{{{I}_{v}}}{{{N}_{h}}}{{S}_{h}}-\beta \left( \frac{{{I}_{s}}+\tau {{I}_{a}}}{{{N}_{h}}} \right) {{S}_{h}}-d{{S}_{h}}, \\ \displaystyle \frac{d{{E}_{h}}}{dt}=bc\frac{{{I}_{v}}}{{{N}_{h}}}{{S}_{h}}+\beta \left( \frac{{{I}_{s}}+\tau {{I}_{a}}}{{{N}_{h}}} \right) {{S}_{h}}-\left( \delta +d \right) {{E}_{h}},\\ \displaystyle \frac{d{{I}_{{s}}}}{dt}=\phi \delta {{E}_{h}}-\left( \eta +d \right) {{I}_{{s}}},\\ \displaystyle \frac{d{{I}_{a}}}{dt}=\left( 1-\phi \right) \delta {{E}_{h}}-\left( \eta +d \right) {{I}_{a}},\\ \displaystyle \frac{d{{R}_{h}}}{dt}=\eta \left( {{I}_{{s}}}+{{I}_{a}} \right) -d{{R}_{h}},\\ \displaystyle \frac{d{{A}_{v}}}{dt}=\theta \left( 1-\frac{{{A}_{v}}+{{J}_{v}}}{K} \right) {{N}_{v}}-p\left( \frac{{{I}_{s}}+q{{I}_{a}}}{{{N}_{h}}} \right) {{A}_{v}}-\left( \alpha +f \right) {{A}_{v}},\\ \displaystyle \frac{d{{J}_{v}}}{dt}=p\left( \frac{{{I}_{s}}+q{{I}_{a}}}{{{N}_{h}}} \right) {{A}_{v}}-\left( \alpha +f \right) {{J}_{v}},\\ \displaystyle \frac{d{{S}_{v}}}{dt}=\alpha {{A}_{v}}-ac\left( \frac{{{I}_{s}}+k{{I}_{a}}}{{{N}_{h}}} \right) {{S}_{v}}-\mu {{S}_{v}},\\ \displaystyle \frac{d{{E}_{v}}}{dt}=ac\left( \frac{{{I}_{s}}+k{{I}_{a}}}{{{N}_{h}}} \right) {{S}_{v}}-\left( \gamma +\mu \right) {{E}_{v}},\\ \displaystyle \frac{d{{I}_{v}}}{dt}=\gamma {{E}_{v}}+\alpha {{J}_{v}}-\mu {{I}_{v}}.\\ \end{array} \right. \end{aligned}$$

Detailed explanations of the variable and parameters are shown in Table 1. All the variables and parameters are non-negative. Similar to the proofs in^21,22^, it is obtained that the following $\Omega$ is positively invariant and attracting under the flow described by the system (1).

$$\begin{aligned} \Omega= & {} \left\{ {\left( {{S_h},{E_h},{I_\mathrm{{s}}},{I_a},{R_h},{A_v},{J_v},{S_v},{E_v},{I_v}} \right) \in R_ + ^{10}} \right. |{S_h},{E_h},{I_\mathrm{{s}}},{I_a},{R_h},{A_v},{J_v},{S_v},{E_v},{I_v} \ge 0, \\{} & {} \left. {{S_h} + {E_h} + {I_\mathrm{{s}}} + {I_a} + {R_h} \le {N_h},{A_v}\mathrm{{ + }}{J_v} \le {\alpha K/\mu },{S_v} + {E_v} + {I_v} \le {N_v}} \right\} . \end{aligned}$$

Temperature, as the most important factor in the modulation of mosquito-borne diseases, is considered by including in the model parameters, where the parameters are functions of temperature *T*. Based on observations from laboratory studies^23–25^, they are presented below.

2 $$\begin{aligned} \alpha \left( T \right){} & {} = 7\left( 0.131-0.05723T + 0.01164{{T}^{2}} -0.001341{{T}^{3}} +0.00008723{{T}^{4}} \right. - 0.000003017{{T}^{5}} \nonumber \\{} & {} \quad \left. +5.153\times {{10}^{-8}}{{T}^{6}} -3.42\times {{10}^{-10}}{{T}^{7}} \right) , \end{aligned}$$

3 $$\begin{aligned} f\left( T \right){} & {} =7\left( 2.13-0.3787T+0.02457{{T}^{2}}-0.0006778{{T}^{3}}+0.000006794{{T}^{4}} \right) , \end{aligned}$$

4 $$\begin{aligned} \mu \left( T \right){} & {} = 7\left( {{{0}}.{{8692 - 0}}.{{159}}T{{ + 0}}.{{01116}}{T^{{2}}}{{ - 0}}.{{0003408}}{T^{{3}}}{{ + 0}}.{{000003809}}{T^{{4}}}} \right) , \end{aligned}$$

5 $$\begin{aligned} \theta \left( T \right){} & {} = 7\left( -5.4+1.8T-0.2124{{T}^{2}}+0.01015{{T}^{3}}-0.0001515{{T}^{4}} \right) , \end{aligned}$$

6 $$\begin{aligned} a\left( T \right){} & {} = \max \left\{ {{{0}}{{.001044}}T\left( {T - {{12}}{{.286}}} \right) {{\left( {32.461 - T} \right) }^{1/2}},0} \right\} , \end{aligned}$$

7 $$\begin{aligned} \gamma \left( T \right){} & {} = 7/ \left( {{{4 + exp}}\left( {{{5}}{{.15}} - {{0}}{{.123}}T} \right) } \right) , \end{aligned}$$

8 $$\begin{aligned} c\left( T \right){} & {} = 7 \left( {{{0}}{{.0043}}T{{ + 0}}{{.0943}}} \right) . \end{aligned}$$

**Table 1:** Description of parameters in the model (the time unit is week or per week).

Parameters	Definitions	Value	Source
*a*	Human-to-vector transmission probability per bite	(6)	^23,25^
*b*	Vector-to-human transmission probability per bite	[a]	
*c*	Number of bites per mosquito per day	(8)	^23^
$\theta$	Intrinsic oviposition rate of adult mosquitoes	(5)	^23^
*K*	Environmental capacity of larval mosquitoes	[c]	Estimation
*k*	Relative (asymptomatic) human-to-vector transmissibility	0.5	^26,27^
$\beta$	Human-to-human transmission rate	[b]	Estimation
$\tau$	Relative (asymptomatic) human-to-human transmissibility	0.5	^26,27^
*p*	Transmission rate from infected person fecal to larval	[b]	Estimation
*q*	Relative (asymptomatic) human-to-larva transmissibility	0.5	^26,27^
$\alpha$	Aquatic transition rate to adult stage	(2)	^23^
$\phi$	Proportion of symptomatical infections	0.18	^28^
$1/\delta$	Intrinsic incubation period in human	0.5	^29^
$1/\eta$	Duration of the human infection period	2	^30^
$1/\gamma$	External incubation period of mosquito	(7)	^23^
1/*d*	Human life span	3950	
${1}/{f}\;$	Lifespan of aquatic mosquitoes	(3)	^23,24^
${1}/{\mu }\;$	Lifespan of adult mosquitoes	(4)	^23,24^

[a] *b* is determined by the effective reproduction number $R_{t}$ in the case study.

[b] $\beta$ and *p* are estimated using the MCMC approach in the case study.

[c] *K* is assumed to be proportional to population size, i.e.,*K* = $\varphi {N}_{h}$, where $\varphi$ is estimated by MCMC method.

## Mathematical analysis

The transmission dynamics of the proposed model are analyzed mathematically, in which the epidemic threshold (i.e., the basic reproduction number) is calculated by the next generation matrix method, and the evolution trends of the model solutions are investigated by stability theory.

### Basic reproduction number

The basic reproduction number ${{R}_{0}}$ is interpreted as the average number of secondary cases that are produced by a single primary case in a fully susceptible population, acting as the critical measure of the transmissibility^31^. The basic reproduction number ${{R}_{0}}$ is calculated by using the theory of next generation matrix^31^. It is written as^31^: ${{R}_{0}}=\rho \left( F{{V}^{-1}} \right)$, where *F* is the rate of occurring new infections, and *V* is the rate of transferring individuals outside the original group. Here $\rho$ represents the spectral radius of matrix.

Let the right hand side of the system (1) equal to zero, it is obtained the disease-free equilibrium of the model

$$\begin{aligned} {{E}_{0}}=\left( S_{h}^{0},E_{h}^{0},I_{s}^{0},I_{a}^{0},R_{h}^{0},A_{v}^{0},J_{v}^{0},S_{v}^{0},E_{v}^{0},I_{v}^{0} \right) {=}\left( N_{h},0,0,0,0,M_{v}^{*},0,N_{v}^{*},0,0 \right) , \end{aligned}$$

where

$$\begin{aligned} M_{v}^{*}=\frac{K\left( \alpha \theta -\mu \left( \alpha +f \right) \right) }{\alpha \theta },N_{v}^{*}=\frac{K\left( \alpha \theta -\mu \left( \alpha +f \right) \right) }{\mu \theta }. \end{aligned}$$

It follows from the next generation matrix method^31^ that

$$\begin{aligned} F = \left( {\begin{array}{*{20}{c}} 0 &{} \beta &{} {\beta \tau } &{} 0 &{} 0 &{} {bc} \\ 0 &{} 0 &{} 0 &{} 0 &{} 0 &{} 0 \\ 0 &{} 0 &{} 0 &{} 0 &{} 0 &{} 0 \\ 0 &{} {\frac{{pM_v^{{*}}}}{{{N_h}}}} &{} {\frac{{pqM_v^\mathrm{{*}}}}{{{N_h}}}} &{} 0 &{} 0 &{} 0 \\ 0 &{} {\frac{{acN_v^*}}{{{N_h}}}} &{} {\frac{{ackN_v^*}}{{{N_h}}}} &{} 0 &{} 0 &{} 0 \\ 0 &{} 0 &{} 0 &{} 0 &{} 0 &{} 0 \\ \end{array}} \right) , V = \left( {\begin{array}{*{20}{c}} {\delta \! +\! d} &{} 0 &{} 0 &{} 0 &{} 0 &{} 0 \\ {\! -\! \phi \delta } &{} {\eta \! +\! d} &{} 0 &{} 0 &{} 0 &{} 0 \\ {\! -\! \left( {1 \!-\! \phi } \right) \delta } &{} 0 &{} {\eta \!+\! d} &{} 0 &{} 0 &{} 0 \\ 0 &{} 0 &{} 0 &{} {\alpha \!+\! f} &{} 0 &{} 0 \\ 0 &{} 0 &{} 0 &{} 0 &{} {\gamma \! +\! \mu } &{} 0 \\ 0 &{} 0 &{} 0 &{} { \!-\! \alpha } &{} { \!-\! \gamma } &{} \mu \\ \end{array}} \right) . \end{aligned}$$

Using ${{R}_{0}}=\rho \left( F{{V}^{-1}} \right)$^31^, the basic reproduction number is calculated as follows:

9 $$\begin{aligned} {{R}_{0}}=\frac{{{R}_{h{{h}_{1}}}}+\sqrt{R_{h{{h}_{1}}}^{2}+4R_{h{{v}_{1}}}^{2}}}{2}, \end{aligned}$$

where

$$\begin{aligned} {{R}_{h{{h}_{1}}}}= & {} \frac{\delta \beta \left( \phi +\left( 1-\phi \right) \tau \right) }{\left( \delta +d \right) \left( \eta +d \right) },\\ {R_{h{v_1}}}= & {} \sqrt{\frac{{pbc\alpha \delta M_v^\mathrm{{*}}\left( {\phi + \left( {1 - \phi } \right) q} \right) \left( {\gamma + \mu } \right) + ba{c^2}\gamma \delta N_v^*\left( {\phi + \left( {1 - \phi } \right) k} \right) \left( {\alpha + f} \right) }}{{{N_h}\mu \left( {\delta + d} \right) \left( {\eta + d} \right) \left( {\gamma + \mu } \right) \left( {\alpha + f} \right) }}}. \end{aligned}$$

Some typical features can be observed from Eq. (9): (a) ${{R}_{h{{h}_{1}}}}$ and ${{R}_{h{{v}_{1}}}}$ represent the parts of the basic reproductive number ${{R}_{0}}$ contributed by sexual transmission and mosquito-borne transmission, respectively; (b) ${{R}_{h{{h}_{1}}}}$ is determined by human-to-human transmission; (c) ${{R}_{h{{v}_{1}}}}$ is determine by parameters related to mosquitoes and humans, which can be further divided into two stages of larval transmission and adult transmission.

### Model stability

Mosquitoes breeding in contaminated water can become infected with ZIKV, which can shorten the virus transmission cycle and promote the spread of ZIKV^14^. Based on this, the following mathematical analysis is divided into two cases, with or without larval infection in the virus sewage, corresponding to $p=0$ or $p\ne 0$.

#### $p=0$ (without larval mosquito infection in the virus sewage)

In this case there is no larval infection ${{J}_{v}}$ item in the system (1). The basic reproduction number of the available model is

10 $$\begin{aligned} {{\overline{R}_{0}}}=\frac{{{R}_{h{{h}_{2}}}}+\sqrt{R_{h{{h}_{2}}}^{2}+4R_{h{{v}_{2}}}^{2}}}{2}, \end{aligned}$$

where

$$\begin{aligned} {R_{h{h_\mathrm{{2}}}}} = \frac{{\delta \beta \left( {\phi + \left( {1 - \phi } \right) \tau } \right) }}{{\left( {\delta + d} \right) \left( {\eta + d} \right) }},{R_{h{v_\mathrm{{2}}}}} = \sqrt{\frac{{ba{c^2}\delta \gamma N_v^*\left( {\phi + \left( {1 - \phi } \right) k} \right) }}{{{N_h}\mu \left( {\delta + d} \right) \left( {\eta + d} \right) \left( {\gamma + \mu } \right) }}}. \end{aligned}$$

##### Theorem 3.1

*When* $p=0$, *if* ${{\overline{R}_{0}}}<1$, *then the disease-free equilibrium of the system* (1) *is locally asymptotically stable.*

The proof is similar to that of Theorem 3.5, so it is omitted here.

The expression of endemic equilibrium is denoted by

$$\begin{aligned} E_0^* = \left( {S_h^\mathrm{{*}},E_h^\mathrm{{*}},I_s^\mathrm{{*}},I_a^\mathrm{{*}},R_h^\mathrm{{*}},A_v^\mathrm{{*}},S_v^\mathrm{{*}},E_v^*,I_v^*} \right) . \end{aligned}$$

Letting the right-hand side of system (1) to be zeros, direct calculation yields an equation about infectivity $\lambda _{h}^{*}$ as

$$\begin{aligned} \lambda _{h}^{*}=bc\frac{{{I}_{v}^{*}}}{{{N}_{h}}}+\beta \left( \frac{{{I}_{s}^{*}}+\tau {{I}_{a}^{*}}}{{{N}_{h}}} \right) . \end{aligned}$$

Let $${{a}_{1}}=\alpha +f, {{a}_{2}}=\gamma +\mu , {{a}_{3}}=\eta +d, {{a}_{4}}=\delta +d. $$

It is further obtained that

11 $$\begin{aligned} \lambda _h^*\mathrm{{ = }}\frac{{bc\gamma N_v^*{H_1}\lambda _h^*d}}{{{N_h}{a_2}\left( {\mu {a_3}{a_4}\left( {\lambda _h^* + d} \right) + \lambda _h^*d{H_1}} \right) }} + \frac{{\lambda _h^*d{H_3}}}{{{a_3}{a_4}\left( {\lambda _h^* + d} \right) }}, \end{aligned}$$

where ${{H}_{1}}=ac\left( \phi \delta +\left( 1-\phi \right) \delta k \right) ,{{H}_{2}}=p\left( \phi \delta +\left( 1-\phi \right) \delta q \right) ,{{H}_{3}}=\beta \left( \phi \delta +\left( 1-\phi \right) \delta \tau \right)$. It follows from Eq. (11) that

12 $$\begin{aligned} {{c}_{0}}{{\left( \lambda _{h}^{*} \right) }^{2}}+{{c}_{1}}\lambda _{h}^{*}+{{c}_{2}}=0, \end{aligned}$$

where

$$\begin{aligned} {{c}_{0}}= & {} {{N}_{h}}{{a}_{2}}{{a}_{3}}{{a}_{4}}\left( \mu {{a}_{3}}{{a}_{4}}+{{H}_{1}}d \right) >0,\\ {{c}_{1}}= & {} d{{N}_{h}}{{a}_{2}}{{a}_{3}}{{a}_{4}}\left( 2\mu {{a}_{3}}{{a}_{4}} \!+\! {{H}_{1}}d \right) \!-\! bc\gamma N_{v}^{*}{{H}_{1}}d{{a}_{3}}{{a}_{4}} \!-\! dN_{h}{{a}_{2}}{{H}_{3}}\left( \mu {{a}_{3}}{{a}_{4}} \!+\! {{H}_{1}}d \right) ,\\ {{c}_{2}}= & {} \mu N_{h}{{a}_{2}}{{d}^{2}}a_{3}^{2}a_{4}^{2}\left( 1\!-\! \frac{bc\gamma N_{v}^{*}{{H}_{1}}}{\mu {{a}_{2}}{{a}_{3}}{{a}_{4}}N_{h}}\!-\! \frac{{{H}_{3}}}{{{a}_{3}}{{a}_{4}}} \right) . \end{aligned}$$

Here $\lambda _{h}^{*}$ can be obtained by solving the quadratic expression (12), which also determines the expression of the endemic equilibrium. When $\mathop {{{\overline{R}}_{0}}}>1$, one has ${\mathop {{{\overline{R}}_{0}}}}\,R_{h{{v}_{2}}}^{2}+{\mathop {{{\overline{R}}_{0}}}}\,{{R}_{hh2}}>R_{h{{v}_{2}}}^{2}+{\mathop {{{\overline{R}}_{0}}}}\,{{R}_{hh2}}$. It is observed from Eq. (10) that $R_{h{{v}_{2}}}^{2}+{\mathop {{{\overline{R}}_{0}}}}\,{{R}_{hh2}}={\mathop {\overline{R}_{0}^{2}}}$.

Hence $\mathop {{{\overline{R}}_{0}}}>1$ yields $R_{h{{v}_{2}}}^{2}+{{R}_{hh2}}>{\mathop {{{\overline{R}}_{0}}}}>1.$ In this case,

$$\begin{aligned} {c_2} = \mu {N_h}{a_2}{d^2}a_3^2a_4^2\left( {1 - R_{h{v_2}}^2 - {R_{h{h_2}}}} \right)< \mu {N_h}{a_2}{d^2}a_3^2a_4^2\left( {1 - \mathop {{R_0}}\limits ^\_ } \right) < 0. \end{aligned}$$

Since ${{c}_{0}}>0$ and ${{c}_{2}}<0$ (when ${{\overline{R}_{0}}}>1$), it is obtained a unique solutions for Eq. (12), which indicates that the system (1) has a unique endemic equilibrium when ${{\overline{R}_{0}}}>1$. Knowing that ${{c}_{2}}>0$ when $\mathop {{R_0}}\limits ^\_ < 1$, there are three scenarios under this condition:

(i) If ${{c}_{1}}>0$, the symmetry axis of $f\left( \lambda _{h}^{*} \right) ={{c}_{0}}{{\left( \lambda _{h}^{*} \right) }^{2}}+{{c}_{1}}\lambda _{h}^{*}+{{c}_{2}}$ is on the negative half axis of *x*, and there is no intersection point between $f\left( \lambda _{h}^{*} \right)$ and the positive half axis of *x*, so the system (1) has no endemic equilibrium.
(ii) If ${{c}_{1}}<0$ and $c_{1}^{2}=4{{c}_{0}}{{c}_{2}}$, there is only one intersection point between $f\left( \lambda _{h}^{*} \right)$ and the positive half axis of *x*, so the system (1) has a positive equilibrium point.
(iii) If ${{c}_{1}}<0$ and $c_{1}^{2}>4{{c}_{0}}{{c}_{2}}$, $f\left( \lambda _{h}^{*} \right)$ has two intersection points with the positive half axis of *x*, then the system (1) has two positive equilibrium points.

The above analysis concludes the following theorem.

##### Theorem 3.2

*When* $p=0$, *if* ${{\overline{R}_{0}}}>1$, *then* ${{c}_{2}}<0$, *the system* (1) *has a unique endemic equilibrium. If* ${{\overline{R}_{0}}}<1$, *the following conclusions can be drawn*:

(i) *if* ${{c}_{1}}>0$, *then System* (1) *has no endemic equilibrium*;
(ii) *if* ${{c}_{1}}<0$ *and* $c_{1}^{2}=4{{c}_{0}}{{c}_{2}}$, *then System* (1) *has a unique endemic equilibrium*;
(iii) *if* ${{c}_{1}}<0$ *and* $c_{1}^{2}>4{{c}_{0}}{{c}_{2}}$, *then System* (1) *has two endemic equilibria*.

##### Theorem 3.3

*When* $p=0$, *if* ${{\overline{R}_{0}}}=1$ *and* $d{{H}_{1}}{{H}_{3}}>d{{H}_{1}}{{a}_{3}}{{a}_{4}}+a_{3}^{2}a_{4}^{2}\mu$, *then the system* (1) *has a backward bifurcation; if* ${{\overline{R}_{0}}}>1$, *the system* (1) *has a forward bifurcation and the endemic equilibrium is locally asymptotically stable*.

##### *Proof*

$X=\left( {{x}_{1}},{{x}_{2}},{{x}_{3}},{{x}_{4}},{{x}_{5}},{{x}_{6}},{{x}_{7}},{{x}_{8}},{{x}_{9}} \right) ^T$ and $G=\left( g_{1}, g_{2}, g_{3}, g_{4}, g_{5}, g_{6}, g_{7}, g_{8}, g_{9} \right) ^T$ to be the left-hand and right-hand sides of system (1). Thus system (1) can be written as

13 $$\begin{aligned} \frac{dX}{dt}=G. \end{aligned}$$

The Jacobian matrix of system (1) at the disease-free equilibrium ${{E}_{0}}$ is

$$\begin{aligned} J\left( {{E_0}} \right) = \left[ {\begin{array}{*{20}{c}} { - d} &{} 0 &{} { - \beta } &{} { - \beta \tau } &{} 0 &{} 0 &{} 0 &{} 0 &{} { - bc} \\ 0 &{} { - {a_4}} &{} \beta &{} {\beta \tau } &{} 0 &{} 0 &{} 0 &{} 0 &{} {bc} \\ 0 &{} {\phi \delta } &{} { - {a_3}} &{} 0 &{} 0 &{} 0 &{} 0 &{} 0 &{} 0 \\ 0 &{} {\left( {1 - \phi } \right) \delta } &{} 0 &{} { - {a_3}} &{} 0 &{} 0 &{} 0 &{} 0 &{} 0 \\ 0 &{} 0 &{} \eta &{} \eta &{} { - d} &{} 0 &{} 0 &{} 0 &{} 0 \\ 0 &{} 0 &{} 0 &{} 0 &{} 0 &{} { - \left( {\frac{{\theta N_v^*}}{K} + {a_1}} \right) } &{} 0 &{} 0 &{} 0 \\ 0 &{} 0 &{} { - \frac{{acN_v^*}}{{{N_h}}}} &{} { - \frac{{ackN_v^*}}{{{N_h}}}} &{} 0 &{} \alpha &{} { - \mu } &{} 0 &{} 0 \\ 0 &{} 0 &{} {\frac{{acN_v^*}}{{{N_h}}}} &{} {\frac{{ackN_v^*}}{{{N_h}}}} &{} 0 &{} 0 &{} 0 &{} { - {a_2}} &{} 0 \\ 0 &{} 0 &{} 0 &{} 0 &{} 0 &{} 0 &{} 0 &{} \gamma &{} { - \mu } \\ \end{array}} \right] . \end{aligned}$$

The probability of mosquito-to-human transmission *b* is selected as the branching parameter. When ${{\overline{R}_{0}}}=1$, the critical value *b* is solved,

$$\begin{aligned} b={{b}^{*}}=\frac{{{N}_{h}}\mu {{a}_{2}}\left( {{a}_{3}}{{a}_{4}}-{{H}_{3}} \right) }{c\gamma N_{v}^{*}{{H}_{1}}}. \end{aligned}$$

Therefore, when $b={{b}^{*}}$, the characteristic equation of the system is

$$\begin{aligned} \left| \lambda E-{{J}_{{{b}^{*}}}}\left( {{E}_{0}} \right) \right| =\lambda {{\left( \lambda +d \right) }^{2}}\left( \lambda +\mu \right) \left( \lambda +\frac{\theta {{N}_{v}^{*}}}{K}+{a}_{1} \right) \left( \lambda +{{a}_{3}} \right) h\left( \lambda \right) , \end{aligned}$$

where $h(\lambda )={{\lambda }^{3}}+{{b}_{1}}{{\lambda }^{2}}+{{b}_{2}}\lambda +{{b}_{3}}$, with ${{b}_{1}}=\mu +{{a}_{2}}+{{a}_{3}}+{{a}_{4}}$, ${{b}_{2}}=\mu {{a}_{2}}+\mu {{a}_{3}}+\mu {{a}_{4}}+{{a}_{2}}{{a}_{3}}+{{a}_{2}}{{a}_{4}}+{{a}_{3}}{{a}_{4}}-{{H}_{3}}$, and ${{b}_{3}}=\mu {{a}_{2}}{{a}_{3}}+\mu {{a}_{2}}{{a}_{4}}+\mu {{a}_{3}}{{a}_{4}}+{{a}_{2}}{{a}_{3}}{{a}_{4}}-{{H}_{3}}\left( \mu +{{a}_{2}} \right)$. It follows from ${{\overline{R}_{0}}}=1$ that

$$\begin{aligned} \frac{{{H}_{3}}}{2{{a}_{3}}{{a}_{4}}}+\frac{{{H}_{3}}}{2{{a}_{3}}{{a}_{4}}}=\frac{{{H}_{3}}}{{{a}_{3}}{{a}_{4}}}<1. \end{aligned}$$

Considering the coefficients of $h\left( \lambda \right)$, it is clear that ${{b}_{1}}=\mu +{{a}_{2}}+{{a}_{3}}+{{a}_{4}}>0,$ ${{b}_{2}}=\mu {{a}_{2}}+\mu {{a}_{3}}+\mu {{a}_{4}}+{{a}_{2}}{{a}_{3}}+{{a}_{2}}{{a}_{4}}+{{a}_{3}}{{a}_{4}}-{{H}_{3}}>0,$ ${{b}_{3}}=\mu {{a}_{2}}{{a}_{3}}+\mu {{a}_{2}}{{a}_{4}}+\mu {{a}_{3}}{{a}_{4}}+{{a}_{2}}{{a}_{3}}{{a}_{4}}-{{H}_{3}}\left( \mu +{{a}_{2}} \right)>\mu {{a}_{2}}{{a}_{3}}+\mu {{a}_{2}}{{a}_{4}}+\mu {{a}_{3}}{{a}_{4}}+{{a}_{2}}{{a}_{3}}{{a}_{4}}-\left( \mu +{{a}_{2}} \right) {{a}_{3}}{{a}_{4}}>0,$ and ${b_1}{b_2} \!-\! {b_3} = \mu \left( {\mu \!+\! {a_2} \!+\! {a_3} \!+\! {a_4}} \right) \left( {{a_2} \!+\! {a_3} \!+\! {a_4}} \right) \mathrm{{ \!+\! }}\left( {{a_3} \!+\! {a_4}} \right) \left( {a_2^2\mathrm{{ \!+\! }}{a_2}{a_3} \!+\! {a_2}{a_4}\mathrm{{ \!+\! }}{a_3}{a_4} \!-\! {H_3}} \right) > 0$. Hence, it follows from $Rrouth-Hurwitz$ theorem that the real parts of the characteristic roots of the equation $h\left( \lambda \right) =0$ are all negative.

Furthermore, by calculating the eigenvalue of the matrix ${{J}_{{{b}^{*}}}}\left( {{E}_{0}} \right)$, it is found that ${{J}_{{{b}^{*}}}}\left( {{E}_{0}} \right)$ has a zero eigenvalue, and other eigenvalues have negative real parts. Therefore, the system (13) satisfies the conditions of the Central Manifold theorem. The right eigenvector and the left eigenvector corresponding to the eigenvalues $\lambda =0$ of the matrix ${{J}_{{{b}^{*}}}}\left( {{E}_{0}} \right)$ are respectively denoted as

$$\begin{aligned} w={{\left( {{w}_{1}},{{w}_{2}},{{w}_{3}},{{w}_{4}},{{w}_{5}},{{w}_{6}},{{w}_{7}},{{w}_{8}},{{w}_{9}} \right) }^T},v=\left( {{v}_{1}},{{v}_{2}},{{v}_{3}},{{v}_{4}},{{v}_{5}},{{v}_{6}},{{v}_{7}},{{v}_{8}},{{v}_{9}} \right) , \end{aligned}$$

where $${{w}_{1}}=-{a}_{4}{{w}_{2}}/d$$, $${{w}_{2}}>0$$, $${{w}_{3}}=\phi \delta {{w}_{2}}/{a}_{3}$$, $${{w}_{4}}=( 1-\phi )\delta {{w}_{2}}/{a}_{3}$$, $${{w}_{5}}=\eta \delta {{w}_{2}}/(d{a}_{3})$$, $${{w}_{6}}=0$$, $${{w}_{7}}= {a}_{2}{w}_{8}/\mu $$, $${{w}_{8}} = \mu {w}_{9}/\gamma $$, $${{w}_{9}}= {w}_{2}({a}_{4}- {H}_{3}/a_{3})/(bc)$$, and $${{v}_{1}}=0$$, $${{v}_{2}}>0$$, $${{v}_{3}}=(\beta {{v}_{2}}+ac{{N}_{v}^{*}}{{v}_{8}/{N}_{h}} )/{a}_{3}$$, $${{v}_{4}}=(\beta \tau {{v}_{2}}+ack{{N}_{v}^{*}}{{v}_{8}/{N}_{h}} )/{a}_{3}$$, $${{v}_{5}}={{v}_{6}}={{v}_{7}}=0$$, $${{v}_{8}}= \gamma {{v}_{9}}/{a}_{2}$$, $${{v}_{9}}=b^{*}c{{v}_{2}}/\mu $$.

According to Castillo-Chavez and Song theorem^32^, it is calculated

$$\begin{aligned} A= & {} \sum \limits _{i,j,k=1}^{9}{{{v}_{k}}}{{w}_{i}}{{w}_{j}}\frac{{{\partial }^{2}}{{g}_{k}}\left( 0,0 \right) }{\partial {{x}_{i}}\partial {{x}_{j}}}\qquad \\= & {} \sum \limits _{i,j{=}1}^{9}{{{v}_{2}}}{{w}_{i}}{{w}_{j}}\frac{{{\partial }^{2}}{{g}_{2}}\left( 0,0 \right) }{\partial {{x}_{i}}\partial {{x}_{j}}}{+}\sum \limits _{i,j=1}^{9}{{{v}_{8}}}{{w}_{i}}{{w}_{j}}\frac{{{\partial }^{2}}{{g}_{8}}\left( 0,0 \right) }{\partial {{x}_{i}}\partial {{x}_{j}}}\qquad \\= & {} 2{{v}_{2}}w_{2}^{2}\frac{d{{H}_{1}}{{H}_{3}}-d{{H}_{1}}{{a}_{3}}{{a}_{4}}-a_{3}^{2}a_{4}^{2}\mu }{d{{N}_{h}}a_{3}^{2}\mu },\qquad \\ B= & {} \sum \limits _{i,k=1}^{9}{{{v}_{k}}}{{w}_{i}}\frac{{{\partial }^{2}}{{g}_{k}}\left( 0,0 \right) }{\partial {{x}_{i}}\partial {{b}^{*}}}={{v}_{2}}{{w}_{2}}\frac{\gamma cN_{v}^{*}{{H}_{1}}}{\mu {{a}_{2}}{{a}_{3}}{{N}_{h}}}>0. \end{aligned}$$

The sign of *B* will determine the occurrence of a backward bifurcation in a given model. When $b={{b}^{*}}$, the positive and negative of the coefficient *A* determines the local dynamical properties of the disease-free equilibrium^32^. Hence, it follows from Castillo-Chavez and Song theorem that system (1) undergoes a backward bifurcation at ${{\overline{R}_{0}}}=1$ if $d{{H}_{1}}{{H}_{3}}>d{{H}_{1}}{{a}_{3}}{{a}_{4}}+a_{3}^{2}a_{4}^{2}\mu$. When ${{\overline{R}_{0}}}>1$ and close to 1, it has $A<0$ and thus the system has a forward branch and the endemic equilibrium is locally asymptotically stable. $\square$

##### Theorem 3.4

*When* $p=0$, *if* ${{\overline{R}_{0}}}>1,$ *the endemic equilibrium of system* (1) *is globally asymptotically stable.*

##### *Proof*

Define the following functions

14 $$\begin{aligned} {{Q}_{1}}= & {} {{S}_{h}}-S_{h}^{*}-S_{h}^{*}\ln (S_{h}/{S}_{h}^{*})+{{E}_{h}}-E_{h}^{*}-E_{h}^{*}\ln (E_{h}/{E}_{h}^{*}), \end{aligned}$$

15 $$\begin{aligned} {{Q}_{2}}= & {} {{I}_{s}}-I_{s}^{*}-{{I}_{s}^{*}}\ln ({{I}_{s}}/I_{s}^{*}), \end{aligned}$$

16 $$\begin{aligned} {{Q}_{3}}= & {} {{I}_{a}}-I_{a}^{*}-{{I}_{a}^{*}}\ln ({{I}_{a}}/I_{a}^{*}), \end{aligned}$$

17 $$\begin{aligned} {{Q}_{4}}= & {} {{S}_{v}}-S_{v}^{*}-S_{v}^{*}\ln (S_{v}/{S}_{v}^{*})+{{E}_{v}}-E_{v}^{*}-E_{v}^{*}\ln (E_{v}/{E}_{v}^{*}), \end{aligned}$$

18 $$\begin{aligned} {{Q}_{5}}= & {} {{I}_{v}}-I_{v}^{*}-{{I}_{v}^{*}}\ln ({{I}_{v}}/I_{v}^{*}). \end{aligned}$$

Using the inequality $1-x+\ln x\le 0$, for $x>0$, differentiation yields:

$$\begin{aligned}{} & {} Q_{1}^{'} \le \frac{{\beta I_s^*S_h^*}}{{{N_h}}}\left( {\frac{{{I_s}}}{{I_s^*}} - \ln \frac{{{I_s}}}{{I_s^*}} - \frac{{{E_h}}}{{E_h^*}} + \ln \frac{{{E_h}}}{{E_h^*}}} \right) + \frac{{\beta \tau I_a^*S_h^*}}{{{N_h}}}\left( {\frac{{{I_a}}}{{I_a^*}} - \ln \frac{{{I_a}}}{{I_a^*}} - \frac{{{E_h}}}{{E_h^*}} + \ln \frac{{{E_h}}}{{E_h^*}}} \right) \\{} & {} \qquad +\frac{bcI_{v}^{*}S_{h}^{*}}{N_{h}}\left( \frac{{{I}_{v}}}{I_{v}^{*}}-\ln \frac{{{I}_{v}}}{I_{v}^{*}}-\frac{{{E}_{h}}}{E_{h}^{*}}+\ln \frac{{{E}_{h}}}{E_{h}^{*}} \right) \\{} & {} \quad =:{{a}_{1,2}}{{G}_{1,2}}+{{a}_{1,3}}{{G}_{1,3}}+{{a}_{1,5}}{{G}_{1,5}}.\\{} & {} Q_{2}^{'} \le \phi \delta E_{h}^{*}\left( \frac{{{E}_{h}}}{E_{h}^{*}}-\ln \frac{{{E}_{h}}}{E_{h}^{*}}-\frac{{{I}_{s}}}{I_{s}^{*}}+\ln \frac{{{I}_{s}}}{I_{s}^{*}} \right) \\{} & {} \quad =:{{a}_{2,1}}{{G}_{2,1}}.\\{} & {} Q_{3}^{'} \le (1-\phi ) \delta E_{h}^{*}\left( \frac{{{E}_{h}}}{E_{h}^{*}}-\ln \frac{{{E}_{h}}}{E_{h}^{*}}-\frac{{{I}_{a}}}{I_{a}^{*}}+\ln \frac{{{I}_{a}}}{I_{a}^{*}} \right) \\{} & {} \quad =:{{a}_{3,1}}{{G}_{3,1}}.\\{} & {} Q_{4}^{'} \le \frac{acI_{s}^{*}S_{v}^{*}}{N_{h}}\left( \frac{{{I}_{s}}}{I_{s}^{*}}-\ln \frac{{{I}_{s}}}{I_{s}^{*}}-\frac{{{E}_{v}}}{E_{v}^{*}}+\ln \frac{{{E}_{v}}}{E_{v}^{*}} \right) + \frac{ackI_{a}^{*}S_{v}^{*}}{N_{h}}\left( \frac{{{I}_{a}}}{I_{a}^{*}}-\ln \frac{{{I}_{a}}}{I_{a}^{*}}-\frac{{{E}_{v}}}{E_{v}^{*}}+\ln \frac{{{E}_{v}}}{E_{v}^{*}} \right) \\{} & {} \quad =:{{a}_{4,2}}{{G}_{4,2}}+{{a}_{4,3}}{{G}_{4,3}}.\\{} & {} Q_{5}^{'} \le \gamma E_{v}^{*}\left( \frac{{{E}_{v}}}{E_{v}^{*}}-\ln \frac{{{E}_{v}}}{E_{v}^{*}}-\frac{{{I}_{v}}}{I_{v}^{*}}+\ln \frac{{{I}_{v}}}{I_{v}^{*}} \right) \\{} & {} \quad =:{{a}_{5,4}}{{G}_{5,4}}. \end{aligned}$$

**Figure 2:**
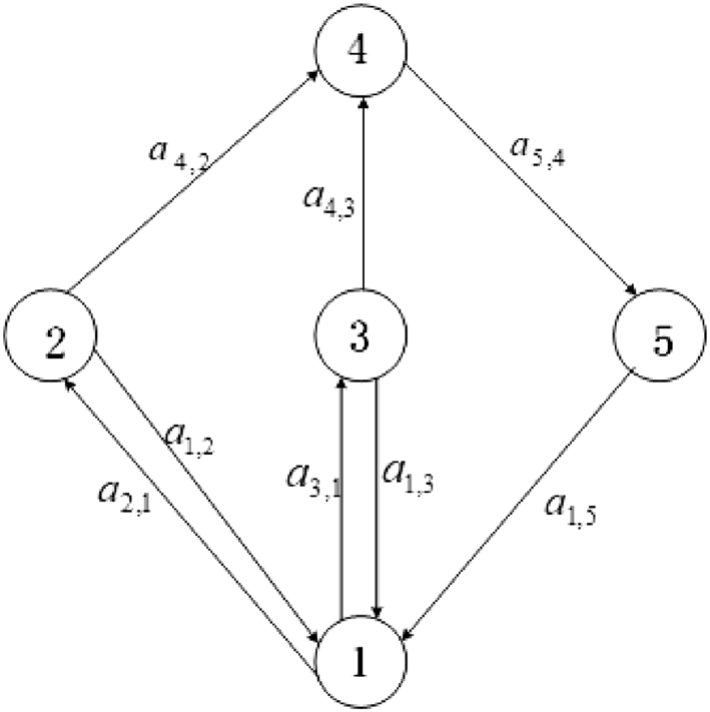
System directed graph.

With the constants ${{a}_{ij}}$ above and $A=[{{a}_{ij}}]$, we construct the (strongly connected) directed graph $\Gamma (A)$ in Fig. 2. If and only if ${{a}_{ij}}>0$, there is a weighted arc$\left( i,j \right)$. Along each of the cycles on the graph, one can verify that $\sum {{{G}_{ij}}=0}$, for instance, ${G_{5,4}}\mathrm{{ + }}{G_{1,5}}\mathrm{{ + }}{G_{2,1}}\mathrm{{ + }}{G_{4,2}}\mathrm{{ = }}0$, and so on. Then, by Theorem 3.5 in^33^, there exist constants ${{c}_{i}}$ such that $Q=\sum {_{i}{{c}_{i}}{{Q}_{i}}}$ is a Lyapunov function for system (1). To find the constants ${{c}_{i}}$, we use the combinatorial identities ${{c}_{i}}{{a}_{ij}}=\sum \nolimits _{k=1}^{p}{{{c}_{j}}}{{a}_{jk}}$ or ${{c}_{i}}{{a}_{ij}}=\sum \nolimits _{k=1}^{p}{{{c}_{k}}}{{a}_{ki}}$. We get ${{c}_{2}}{{a}_{2,1}}={{c}_{1}}{{a}_{1,2}}+{{c}_{4}}{{a}_{4,2}}$, ${{c}_{3}}{{a}_{3,1}}={{c}_{1}}{{a}_{1,3}}+{{c}_{4}}{{a}_{4,3}}$ and ${{c}_{5}}{{a}_{5,4}}={{c}_{1}}{{a}_{1,5}}$. Let ${c_1}\mathrm{{ = }}{c_4}\mathrm{{ = }}1$, and

$$\begin{aligned} {c_2}\mathrm{{ = }}\frac{{\beta I_s^*S_h^*\mathrm{{ + }}acI_s^*S_v^*}}{{{N_h}\phi \delta E_h^*}},{c_3}\mathrm{{ = }}\frac{{\beta \tau I_a^*S_h^*\mathrm{{ + }}ackI_a^*S_v^*}}{{{N_h}\left( {1 - \phi } \right) \delta E_h^*}},{c_5}\mathrm{{ = }}\frac{{bcI_v^*S_h^*}}{{{N_h}\gamma E_v^*}}. \end{aligned}$$

Then the function $Q = {c_1}{Q_1}\mathrm{{ + }}{c_2}{Q_2}\mathrm{{ + }}{c_3}{Q_3}\mathrm{{ + }}{c_4}{Q_4}\mathrm{{ + }}{c_5}{Q_5}$ is a Lyapunov function. Its derivative along the model is

$$\begin{aligned} {{Q}^{'}}= & {} \left( \frac{{{S}_{h}}-S_{h}^{*}}{{{S}_{h}}}S_{h}^{'}+\frac{{{E}_{h}}-E_{h}^{*}}{{{E}_{h}}}E_{h}^{'} \right) {+}{{c}_{2}}\left( \frac{{{I}_{s}}-I_{s}^{*}}{{{I}_{s}}}I_{s}^{'} \right) {+}{{c}_{3}}\left( \frac{{{I}_{a}}-I_{a}^{*}}{{{I}_{a}}}I_{a}^{'} \right) \\{} & {} +\left( \frac{{{S}_{v}}-S_{v}^{*}}{{{S}_{v}}}S_{v}^{'}+\frac{{{E}_{v}}-E_{v}^{*}}{{{E}_{v}}}E_{v}^{'} \right) {+}{{c}_{5}}\left( \frac{{{I}_{v}}-I_{v}^{*}}{{{I}_{v}}}S_{v}^{'} \right) . \end{aligned}$$

Now we consider the set $S=\left\{ x\in R_{+}^{9}:{{Q}^{'}}=0 \right\}$. When ${{Q}^{'}}=0$, one can readily verify that ${{S}_{h}}{=}S_{h}^{*}, {{E}_{h}}{=}E_{h}^{*}, {{I}_{s}}{=}I_{s}^{*}, {{I}_{a}}{=}I_{a}^{*}, {{S}_{v}}=S_{v}^{*}, {{E}_{v}}=E_{v}^{*}$ and ${{I}_{v}}=I_{v}^{*}$. For the left subsystem,

19 $$\begin{aligned} \left\{ \begin{array}{ll} \displaystyle R_{h}^{'}=\eta \left( I_{s}^{*}+I_{a}^{*} \right) -d{{R}_{h}}, \\ \displaystyle A_{v}^{'}=\theta \left( 1-\frac{{{A}_{v}}}{K} \right) N_{v}^{*}-\left( \alpha +f \right) {{A}_{v}}. \end{array} \right. \end{aligned}$$

One can show that system (19) has a unique equilibrium $\left( R_{h}^{*},A_{v}^{*} \right)$, and that this point is globally asymptotically stable for this system. Therefore, the largest and only invariant set in *S* is the endemic equilibrium ${E_{0}^{*}}=\left( S_{h}^{*},E_{h}^{*},I_{s}^{*},I_{a}^{*},R_{h}^{*},A_{v}^{*},S_{v}^{*},E_{v}^{*},I_{v}^{*} \right)$. Using LaSalle¡¯s Invariance Principle, we conclude that the endemic equilibrium $E_{0}^{*}$ globally asymptotically stable in $\Omega$. $\square$

#### $p\ne 0$ (mosquitoes hatched in contaminated water can be infected with ZIKV)

##### Theorem 3.5

*When* ${{R}_{0}}<1$, *the disease-free equilibrium of the system* (1) *is globally asymptotically stable.*

##### *Proof*

Substituting ${{S}_{h}}$, ${{A}_{v}}$ and ${{S}_{v}}$ by ${{N}_{h}}-{{E}_{h}}-{{I}_{a}}-{{I}_{s}}-{{R}_{h}}$, ${{M}_{v}}-{{J}_{v}}$ and ${{N}_{v}}-{{E}_{v}}-{{I}_{v}}$ respectively, it is obtained

$$\begin{aligned} \frac{{d{E_h}}}{{dt}}= & {} \left( {bc\frac{{{I_v}}}{{{N_h}}} + \beta \left( {\frac{{{I_s} + \tau {I_a}}}{{{N_h}}}} \right) } \right) \left( {{N_h} - {E_h} - {I_a} - {I_s} - {R_h}} \right) - \left( {\delta + d} \right) {E_h}, \\ \frac{{d{J_v}}}{{dt}}= & {} p\left( {\frac{{{I_s} + q{I_a}}}{{{N_h}}}} \right) \left( {{M_v} - {J_v}} \right) - \left( {\alpha + f} \right) {J_v} \le p\left( {\frac{{{I_s} + q{I_a}}}{{{N_h}}}} \right) {M_v} - \left( {\alpha + f} \right) {J_v}, \\ \frac{{d{J_v}}}{{dt}}= & {} p\left( {\frac{{{I_s} + q{I_a}}}{{{N_h}}}} \right) \left( {{M_v} - {J_v}} \right) - \left( {\alpha + f} \right) {J_v} \le p\left( {\frac{{{I_s} + q{I_a}}}{{{N_h}}}} \right) {M_v} - \left( {\alpha + f} \right) {J_v}, \\ \frac{{d{E_v}}}{{dt}}= & {} ac\left( {\frac{{{I_s} + k{I_a}}}{{{N_h}}}} \right) \left( {{N_v} - {E_v} - {I_v}} \right) - \left( {\gamma + \mu } \right) {E_v} \le ac\left( {\frac{{{I_s} + k{I_a}}}{{{N_h}}}} \right) {N_v} - \left( {\gamma + \mu } \right) {E_v}. \end{aligned}$$

Let

20 $$\begin{aligned} \left\{ \begin{array}{ll} \displaystyle \frac{d{{\overline{E}}_{h}}}{dt}=bc{\overline{I}_{v}}+\beta \left( {\overline{I}_{s}}+\tau {\overline{I}_{a}} \right) -\left( \delta +d \right) {\overline{E}_{h},} \\ \displaystyle \frac{d{{\overline{I}}_{s}}}{dt}=\phi \delta {\overline{E}_{h}}-\left( \eta +d \right) {\overline{I}_{s},} \\ \displaystyle \frac{d{{\overline{I}}_{a}}}{dt}=\left( 1-\phi \right) \delta {\overline{E}_{h}}-\left( \eta +d \right) {\overline{I}_{a},} \\ \displaystyle \frac{d{{\overline{J}}_{v}}}{dt}=p\left( \frac{{\overline{I}_{s}}+q{\overline{I}_{a}}}{{{N}_{h}}} \right) {{M}_{v}}-\left( \alpha +f \right) {\overline{J}_{v},} \\ \displaystyle \frac{d{{\overline{E}}_{v}}}{dt}=ac\left( \frac{{\overline{I}_{s}}+k{\overline{I}_{a}}}{{{N}_{h}}} \right) {{N}_{v}}-\left( \gamma +\mu \right) {\overline{E}_{v},} \\ \displaystyle \frac{d{{\overline{I}}_{v}}}{dt}=\gamma {\overline{E}_{v}}+\alpha {\overline{J}_{v}-\mu {\overline{I}_{v}}.} \\ \end{array} \right. \end{aligned}$$

It can be seen that the right side of the system (20) is the right side of the matrix $F-V$. According to ${{R}_{0}}=\rho \left( F{{V}^{-1}} \right) <1$, it can be seen that the system (20) has only a balance point $\left( {\overline{E}_{h}},{\overline{I}_{{s}}},{\overline{I}_{a}},{\overline{J}_{v}},{\overline{E}_{v}},{\overline{I}_{v}} \right) {=}\left( 0,0,0,0,0,0 \right)$. Therefore, every non-negative solution of (20) satisfies

$$\begin{aligned} \mathop {\lim }\limits _{t \rightarrow \infty } {{{\bar{E}}}_h}\left( t \right)= & {} 0,\mathop {\lim }\limits _{t \rightarrow \infty } {{{\bar{I}}}_\mathrm{{s}}}\left( t \right) = 0,\mathop {\lim }\limits _{t \rightarrow \infty } {{{\bar{I}}}_a}\left( t \right) = 0, \\ \mathop {\lim }\limits _{t \rightarrow \infty } {{{\bar{J}}}_v}\left( t \right)= & {} 0,\mathop {\lim }\limits _{t \rightarrow \infty } {{{\bar{E}}}_v}\left( t \right) = 0,\mathop {\lim }\limits _{t \rightarrow \infty } {{{\bar{I}}}_v}\left( t \right) = 0. \end{aligned}$$

Because the system (20) is linear, the disease-free equilibrium of the system (20) is globally asymptotically stable.

According to the comparison theorem

$$\begin{aligned}{} & {} E_{h}^{'}\left( t \right) \le \overline{E}_{h}^{'}\left( t \right) , I_{s}^{'}\left( t \right) \le {\overline{I}}_{{{s}}}^{'}\left( t \right) , I_{a}^{'}\left( t \right) \le {\overline{I}}_{{{a}}}^{'}\left( t \right) , \\{} & {} J_{v}^{'}\left( t \right) \le {\overline{J}}_{{{v}}}^{'}\left( t \right) , E_{v}^{'}\left( t \right) \le {\overline{E}}_{{{v}}}^{'}\left( t \right) , I_{v}^{'}\left( t \right) \le {\overline{I}}_{{{v}}}^{'}\left( t \right) . \end{aligned}$$

Thus

$$\begin{aligned} \mathop {\lim }\limits _{t \rightarrow \infty } {E_h}\left( t \right)= & {} 0,\mathop {\lim }\limits _{t \rightarrow \infty } {I_\mathrm{{s}}}\left( t \right) = 0,\mathop {\lim }\limits _{t \rightarrow \infty } {I_a}\left( t \right) = 0,\\ \mathop {\lim }\limits _{t \rightarrow \infty } {J_v}\left( t \right)= & {} 0,\mathop {\lim }\limits _{t \rightarrow \infty } {E_v}\left( t \right) = 0,\mathop {\lim }\limits _{t \rightarrow \infty } {I_v}\left( t \right) = 0. \end{aligned}$$

The disease-free equilibrium is globally attractive, and it is locally stable. So the disease-free equilibrium of the system (1) is globally asymptotically stable. $\square$

##### Theorem 3.6

*When* ${{R}_{0}}>1$, *the system* (1) *exists endemic equilibrium*.

##### *Proof*

It is denoted the expression of endemic equilibrium by

$$\begin{aligned} E_{1}^{*}=\left( S_{h}^{{*}},E_{h}^{{*}},I_{s}^{{*}},I_{a}^{{*}},R_{h}^{{*}},A_{v}^{{*}},J_{v}^{*},S_{v}^{{*}},E_{v}^{*},I_{v}^{*} \right) . \end{aligned}$$

Based on the equilibrium definition, letting the right-hand side of system (1) to be zeros and substituting $E_{1}^{*}$, it is obtained an equation about infectivity $\lambda _{h}^{*}$, $\lambda _{{{v}_{1}}}^{*}$ and $\lambda _{{{v}_{2}}}^{*}$ as

$$\begin{aligned} \lambda _{h}^{*}=bc\frac{{{I}_{v}^{*}}}{{{N}_{h}}}+\beta \left( \frac{{{I}_{s}^{*}}+\tau {{I}_{a}^{*}}}{{{N}_{h}}} \right) , \lambda _{{{v}_{1}}}^{*}=p\left( \frac{{{I}_{s}^{*}}+q{{I}_{a}^{*}}}{{{N}_{h}}} \right) , \lambda _{{{v}_{2}}}^{*}=ac\left( \frac{{{I}_{s}^{*}}+k{{I}_{a}^{*}}}{{{N}_{h}}} \right) . \end{aligned}$$

It follows that

$$\begin{aligned} \lambda _{h}^{*}= & {} \frac{bcN_{v}^{*}}{{{N}_{h}}}\left( \frac{\gamma {{a}_{1}}\lambda _{{{v}_{2}}}^{*}+{{a}_{2}}\left( \mu +\lambda _{{{v}_{2}}}^{*} \right) \lambda _{{{v}_{1}}}^{*}}{{{a}_{2}}\left( \mu +\lambda _{{{v}_{2}}}^{*} \right) \left( {{a}_{1}}+\lambda _{{{v}_{1}}}^{*} \right) } \right) +\frac{\lambda _{h}^{*}d{{H}_{3}}}{{{a}_{3}}{{a}_{4}}\left( \lambda _{h}^{*}+d \right) },\\ \lambda _{{{v}_{1}}}^{*}= & {} \frac{\lambda _{h}^{*}d{{H}_{2}}}{{{a}_{3}}{{a}_{4}}\left( \lambda _{h}^{*}+d \right) },\\ \lambda _{{{v}_{2}}}^{*}= & {} \frac{\lambda _{h}^{*}d{{H}_{1}}}{{{a}_{3}}{{a}_{4}}\left( \lambda _{h}^{*}+d \right) }. \end{aligned}$$

Substituting $\lambda _{{{v}_{1}}}^{*}$ and $\lambda _{{{v}_{2}}}^{*}$ into $\lambda _{h}^{*}$, sorted out

$$\begin{aligned} \lambda _{h}^{*}=\frac{Y}{X}+\frac{\lambda _{h}^{*}d{{H}_{3}}}{{{a}_{3}}{{a}_{4}}\left( \lambda _{h}^{*}+d \right) }, \end{aligned}$$

where

$$\begin{aligned} Y= & {} bc{{N}_{v}^{*}}\lambda _{h}^{*}d\left( \gamma {{a}_{1}}{{a}_{3}}{{a}_{4}}{{H}_{1}}\left( \lambda _{h}^{*}+d \right) \right. \left. +{{a}_{2}}{{H}_{2}}\left( \mu {{a}_{3}}{{a}_{4}}\left( \lambda _{h}^{*}+d \right) +{{H}_{1}}\lambda _{h}^{*}d \right) \right) , \\ X= & {} {{a}_{2}}{{N}_{h}}\left( \mu {{a}_{3}}{{a}_{4}}\left( \lambda _{h}^{*}+d \right) +\lambda _{h}^{*}d{{H}_{1}} \right) \left( \lambda _{h}^{*}d{{H}_{2}}+{{a}_{1}}{{a}_{3}}{{a}_{4}}\left( \lambda _{h}^{*}+d \right) \right) . \end{aligned}$$

Let

$$\begin{aligned} f\left( \lambda _{h}^{*} \right) =\frac{Y}{\lambda _{h}^{*}X}+\frac{d{{H}_{3}}}{{{a}_{3}}{{a}_{4}}\left( \lambda _{h}^{*}+d \right) }-1. \end{aligned}$$

Substituting $\lambda _{h}^{*}$ by 0 and $+\infty$, it follows that $f\left( +\infty \right) =-1<0$, and

$$\begin{aligned} f\left( 0 \right)= & {} \frac{ bcN_{v}^{*}\left( \gamma {{a}_{1}}{{H}_{1}}+{\mu {a}_{2}}{{H}_{2}} \right) }{{{\mu }}{{N}_{h}}{{a}_{1}}{{a}_{2}}{{a}_{3}}{{a}_{4}}}+\frac{{{H}_{3}}}{{{a}_{3}}{{a}_{4}}}-1 \\= & {} \frac{\gamma {{a}_{1}}{{H}_{1}}N_{v}^{*}bc+\alpha {{a}_{2}}{{H}_{2}}{{M}_{v}^{*}}bc}{{{\mu }}N_{h}{{a}_{1}}{{a}_{2}}{{a}_{3}}{{a}_{4}}}+\frac{{{H}_{3}}}{{{a}_{3}}{{a}_{4}}}-1>{{R}_{0}}-1>0. \end{aligned}$$

According to the existence theorem of zero point, when ${{R}_{0}}>1$, there exists $\lambda _{h}^{*}>0$, such that $f\left( \lambda _{h}^{*} \right) =0$, indicating the existence of endemic equilibrium for the system (1). $\square$

## Model application

The massive outbreak of Zika in Brazil during 2015 and 2016 is used as a typical prototype to validate the proposed model. The $\mathrm{{estimate}}\_\mathrm{{R}}$ function in EpiEstim package of *R* language is used to calculate the effective reproduction number $R_{t}$ based on weekly morbidity time series, intergenerational distribution and window period^34^. $R_{t}$ is the average number of people someone infected at time *t* can infect over their entire infectious lifespan, which can quantify the immediate transmissibility. Here $R_{t}$ is used to determine the parameter *b* in the model. Since clinical studies have shown that the viral load of asymptomatically infected patients with Zika is about half that of symptomatic patients, it is assumed that the transmission probability of asymptomatic people is half that of symptomatic people^26,27^. In addition, due to the large temperature difference between the north and south of Brazil, the average weekly temperature $T=24.15\,^\circ$C of the three cities of Manaus, Brasilia and Porto Alegre (divided into the north, central and south of Brazil) is employed in the model parameters expressions.

In order to verify the model of case study, MCMC method is used to quantify the uncertain parameters: $\vartheta$, $\beta$ and *p*. In the absence of surveillance data, it is assumed that the initial values of total mosquitoes sizes are at the positive stable level, ${{M}_{v}}(0)$ and ${{N}_{v}}(0)$ are given by (). The initial values of the numbers of vector in latent and infected states are also estimated by MCMC method. The MCMC is run for 200,000 iterations for each parameter and the posterior distributions are compiled from the final 80% of the iterations. The model is validated with 95% confidence interval of posterior estimations by sampling 1000 times of the posterior distributions of the estimated parameters and by incorporating them into the model. The normalized forward sensitivity and global sensitivity are used to quantify the importance of parameters related to the modeling incidence and the reproduction number $R_{0}$. The normalized forward sensitivity is computed by $\frac{\partial R_{0} }{\partial x}\frac{x}{R_{0}}$ for parameter *x*. The global sensitivity is realized by computing partial rank correlation coefficient (PRCC) based on Latin hypercube sampling for the model inputs and outputs^35^.

### Study area and data collection

Brazil is located in the eastern South America, with longitudes 35$^\circ$ W to 74$^\circ$ W and latitudes 5$^\circ$ N to 35$^\circ$ S. It is the largest country in South America, whose area is about 8,514,900 square kilometers. Brazil has typical tropical climate, with the annual average temperature as 20–28 $^\circ$C. Such climate is suitable for the growth of Aedes mosquitoes. As a result, Brazil faces public health problems related to mosquito-borne diseases.

The study use medical records of human Zika infections reported in Brazil from January 2015 to September 2016. The weekly numbers of cases is collected from the World Health Organization^36^ and the Brazilian Ministry of Health, which is shown in Table S2 (see Supplementary Information [SI]). Brazilian population data is obtained from the Brazilian Institute of Statistics (https://www.ibge.gov.br/). Brazil’s weekly average temperature record is extracted from the Brazilian Meteorological Agency (https://previsao.inmet.gov.br/).

### Result

**Figure 3:**
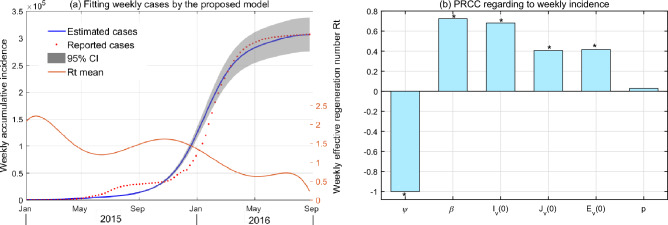
(**a**) The fitting results of the ZIKV cases in Brazil from the proposed model. The light colored area is the 95% confidence intervals (CIs) for all 1000 simulations. (**b**) Global sensitivity analysis for weekly incidence, where the PRCCs are the mean values in each week, and $$*$$ indicates a significant difference from zero (with p value $$ < 0.01$$).

The reporting data shows that Brazil witnessed the first human infection in January 2015, but few case is recorded in the following 2 months. ZIKV began to expand since April, with the first epidemic peak in early July. After a continuous low incidence, human infections reached a higher peak in February 2016 and then decrease rapidly until zero in September. By fitting the epidemic curve of the cumulative number of weekly cases by the model, the estimated results of unknown parameters are shown in Table S1 and Fig. S1. As shown in Fig. 3a, it is observed that the estimated parameters enable the model to draw a good fitting capacity, except the first peak. The fitting deviation is possibly due to the temporal heterogeneity of transmission parameters and detection efficiency of human cases. Uncertainty analysis indicates that the model is robust in exploring transmission dynamics, which can draw consistent evolution of weekly accumulate incidence in case of random sampling (see Fig. S1 in SI).

Sensitivity analysis is used to quantify the response of model outputs to parameter variation. Results from Fig. 3 (b) indicate that the most sensitive parameter to the modeling infection is environmental capacity rate of mosquitoes ($\varphi$), followed by the human-to-human transmission rate ($\beta$) and the initial value of infected adult mosquitoes (${{I}_{v}}$). Yet the model output is not sensitive to the transmission rate from infected person fecal to larval (*p*).

The contributions of different routes to the ZIKV infection in Brazil is estimated by splitting the values of the basic reproduction number $R_{0}$. Substituting the estimated parameters (in which *b* is chosen as the average value of the previous 7 weeks) into Eq. (9) yields the basic reproduction number to be $R_{0}=2.13$ (95% CI 1.61–2.64), in which the contribution of mosquito transmission is 2.04 (95% CI 1.53–2.55). Moreover, the transmission of the virus by mosquitoes could be divided into adult mosquitoes and larval mosquitoes in sewage, in which the latter contribution to $R_{0}$ is estimated to be 0.48 (95% CI 0.26–0.71).

**Figure 4:**
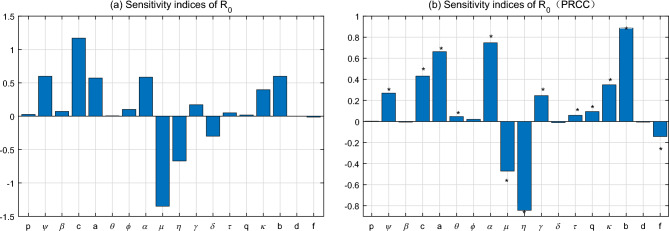
Sensitivity analysis of the basic reproduction number $R_{0}$. (**a**) Normalize forward sensitivity; (**b**) global sensitivity with PRCC.

Results of sensitivity analysis toward $R_{0}$ are presented in Fig. 4. Two different methods of sensitivity analysis show similar results, indicating the robustness of $R_{0}$ to the parameters used. It is observed that the mortality of adult mosquitoes ($\mu$), infection period of human beings ($\eta$), mosquito biting rate (*c*), environmental capacity rate of mosquitoes ($\varphi$), human-mosquito transmission rates (*a* and *b*) and transition rate of mosquitoes from larval to adult ($\alpha$) are most sensitive parameters to determine $R_{0}$. Yet $R_{0}$ is less sensitive to the transmission rates from fecal to larval and from human to human (*p* and $\beta$), oviposition rate of adult mosquitoes ($\theta$) proportion of symptomatical infections ($\phi$), human death rate (*d*) and relative infectivity of asymptomatic infections (*q* and $\tau$). These parameters could play minor roles in causing human infection of ZIKV.

**Figure 5:**
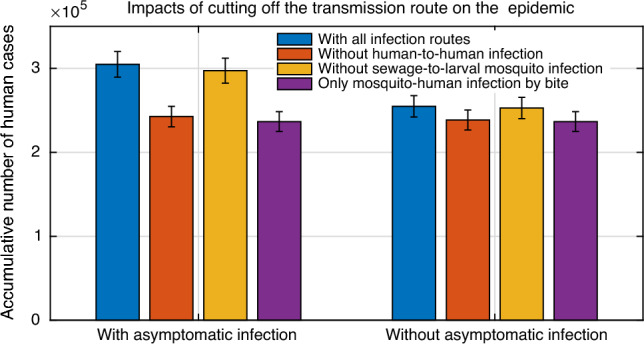
Influence of cutting off different transmission routes on the outbreak of Zika. The cumulative numbers of humans infection in Brazil with 95% CIs are estimated in cases of different transmission routes during January 2015 and September 2016.

Figure 5 shows the effects of different transmission paths of the Zika outbreak in Brazil on the cumulative number of human infections from January 2015 to September 2016. The fitting results demonstrate that the total infections could be 304,648 (95% CI 304,096–305,199). If without human infection by sex or without larvae mosquito infection by sewage, this number could be 242,487 (95% CI 242,025–242,949) or 297,115 (95% CI 296,558–297,674). If without both of the above transmission routs, it could become 236,455 (95% CI 235,994–236,918). Of these three assumptions, the total number of human infections decreased by 20.4%, 2.47% and 22.38%, respectively. Moreover, in the absence of asymptomatic infection, the cumulative number of human infections could be 254,715 (95% CI 254,238–255,192), making human infections decrease by 16.39%. In this case, the above-mention three circumstance lead to the declines of human infections by 6.43%, 0.79% and 7.17%, respectively.

**Figure 6:**
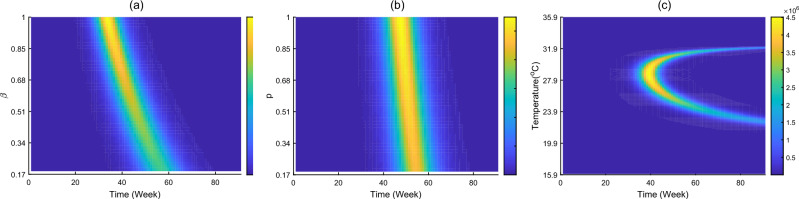
Impacts of (**a**) sexual transmission rate $\beta$, (**b**) sewage transmission rate *p* and (**c**) temperature on the number of new infections cases, which is achieved by simulating the proposed model with other parameters equal to those of the fitting results.

Figure 6 shows the time evolution of human infection in cases of different human-to-human transmission rate $(\beta )$, larval mosquito transmission rate in sewage (*p*) and temperature. It is observed that the increase of transmission rates through sex and sewage would lead to moderately higher number of human cases and slightly faster of disease transmission, Such effect caused by high sex transmission rate is more significant. Furthermore, temperature around $28.7\,^\circ$C is most favourable for ZIKV infection, and temperature away from this value could cause low morbidity and low infection risk.

**Figure 7:**
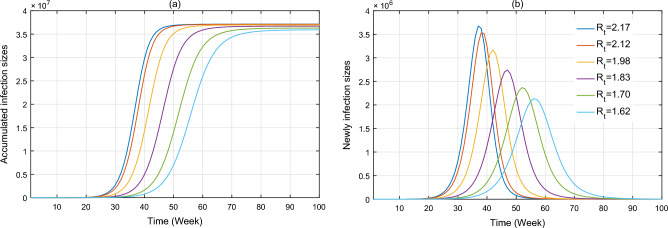
Impacts of different effective reproduction number on the number of human infection in Brazil during January 2015 and November 2016, which is achieved by simulating the proposed model with other parameters equal to those of fitting results.

Figure 7 shows the effectiveness of the implementation of control measures on the spread of ZIKV infection in Brazil during January 2015 and November 2016. The intervention is measured by the effective reproduction number $R_{t}$. Here the values of $R_{t}$ are fixed to be 2.17, 2.12, 1.98, 1.83, 1.70 and 1.62, which are its average values of in the previous 1–5, 1–10, 1–15, 1–20, 1–25 and 1–30 weeks, respectively. The simulation results indicate that (1) if $R_{t}$ = 2.17 (few intervention measures), the cumulative number of human infections could reach 37.2 million, that is over 121 times of reported cases, with early peak of new cases as 3.7 million around the 37th week; (2) if $R_{t}$ = 1.62 (limited intervention measures), the cumulative number of human cases could be 36 million, with peak number of new cases as 2.1 million around the 57th week. It is observed that the decrease of $R_{t}$ causes an evident decline of humans infections and quick arriving of peak infection.

## Discussion

A modeling framework for inferring ZIKV transmission patterns is attempted in this paper. Technologically, a new SEIR-AJ-SEI dynamic model is established, which couples the ZIKV circulation among/between mosquitoes and humans under potential routes, including mosquito bite, sex contact, and sewage breed. Compared with existing ZIKV model, such as using a discrete stochastic SEIR-SEI model to predict the optimal effect of bednets, infection treatment and insecticide spraying on disease transmission^18^, using a SEIIAR-SEI model to infer the impact of mosquito-borne and sexual transmission on ZIKV spread^9^, using a continuing climate-driven SEIIIR-SEI model to study threshold dynamics in a seasonal model of Zika virus disease^19^, using a high-dimensional ODE system to describe the joint dynamics of Zika and dengue^17^, and using partial differential equations model to understand how spatial heterogeneity of the vector and host populations influence ZIKV dynamics^16^, the proposed model is a combined update of recent works, which converges more dynamical detail.

First, the ZIKV transmission dynamics are clarified mathematically. The basic reproduction number $R_{0}$ is calculated by using the next generation matrix, which is found to be determined by all the transmission routes. It is verified that the disease-free equilibrium is locally stable when the associated basic reproduction number is less than unity, and there exits endemic equilibrium when basic reproduction number is larger than unity. If without larval infection in virus sewage, central manifold theorem demonstrates that the system is capable of undergoing the phenomenon of backward bifurcation, under which the stable disease-free equilibrium would co-exist with a stable endemic equilibrium and an unstable endemic equilibrium. Such phenomenon could be caused by the combined effects of multiple transmission routes. The existence of larval infection in virus sewage would cause more complicated transmission dynamics. Dynamic details indicate that great efforts could be needed for preventing ZIKV infection.

Second, the proposed model is further validated to explore more transmission dynamics by fitting the reported cases of ZIKV infection in Brazil from January 2015 to September 2016. Two important insights based on this study may provide scientific clues for evaluating the infection risk and guiding control.

On the one hand, the impact of the transmission routes on the Zika epidemic is evaluated. It is found that mosquito-human infection by bite is still the prominent path for ZIKV occurrence, accounting for 85.7% of the basic reproduction number $R_{0}$, but the contributions by sexual transmission and larval transmission in sewage are 3.5% and 10.8%, respectively, both of which are less than 1. Therefore, these two routes of infection are not enough to trigger a large-scale outbreak of ZIKV, which is consistent with previous studies^9,11,15^. The sensitivity analysis further confirms that ZIKV infection is dominated by the parameters related to mosquito ecology, rather than those parameters related to asymptomatic or human-human transmission. Yet the latter two transmission routes can limitedly accelerate the development of Zika epidemic and prolongs the outbreak time. Such effects would be more significant for higher concentration of ZIKV in sewage and larger probability of human-to-human infection. Therefore, the prevention and control of ZIKV should target at reducing the infection through mosquito bite, and do not ignore the infection through sex and sewage.

On the other hand, the situations of ZIKV transmission in Brazil are evaluated. The present study suggests that multiple modes of transmission and suitable temperature may be responsible for the large outbreak of ZIKV in Brazil in 2015–2016. Nevertheless, the intervention implemented in Brazil plays an important role in controlling ZIKV infections. If without intervention, the number of human infections with ZIKV in Brazil would increase rapidly^37^, and result in more than 37.2 million cases. After the implementation of control measures in May 2015, the number of effective reproduction $R_{t}$ decreased from 1.69 to less than 1, leading to a rapid decline in morbidity. However, human infections began to rebound rapidly in November of the same year, leading to the second peak of cases in Brazil at 59th week. Meanwhile, control measures were intensified, including mobilizing Brazilian army to support community health services^37^, house-to-house visits and the elimination of potential Aedes breeding sites^37^, aerial spraying of the product to kill larvae or adult mosquito^38^, and reduction of breeding sites through drainage of standing water, waste management and education about mosquitoes and personal protection measures^39^. Under these control measures, the incidence and effective reproduction number dropped fast. The present study indicates that such comprehensive and intensified ZIKV control strategies are highly effective in curtailing ZIKV transmission.

The following limitations need to be clarified. First, since the proposed model is only used to fit the surveillance data reported in Brazil, there may be geographical differences in its application to other countries. Second, since it is impossible to obtain the true values of some model parameters, they are extracted from the literature or are estimated by MCMC method. Third, the study dose not include all the underlying factors, such as human mobility and climate (except temperature). However, the model takes into account the most influential factors and incorporates model parameterizations, providing confidence in the model output for future analysis.

### Supplementary Information

Supplementary Information.

## Data Availability

All data generated or analysed during this study are included in this article and its supplementary information files.
