# Supplementary material for: Transmission dynamics of Zika virus with multiple infection routes and a case study in Brazil
Source: Sci Rep. 2024 Mar 28;14:7424. doi: 10.1038/s41598-024-58025-7 (PMC11369273; doi:10.1038/s41598-024-58025-7)
Supplement: Supplementary file 1 — Supplementary Information. [file 41598_2024_58025_MOESM1_ESM.docx]

**Supplementary Information**

# Transmission dynamics of Zika virus with multiple

# infection routes and a case study in Brazil

# Fitting results of model parameters

#
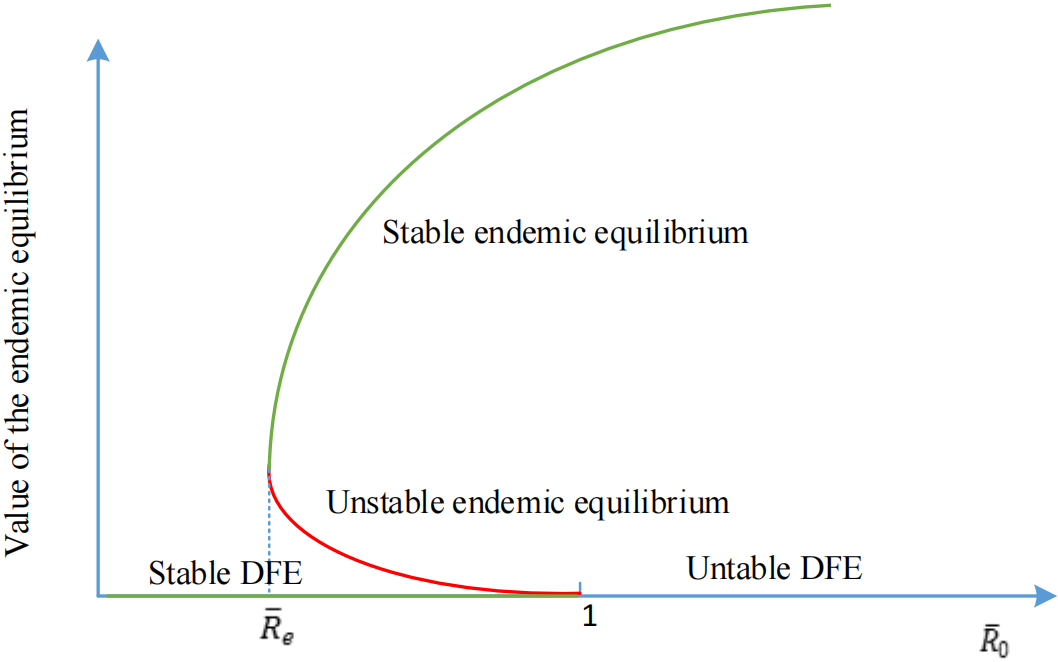


**Figure S1.** The backward bifurcation of the model in case of $p=0$. Here DFE means disease-free equilibrium. $\bar{R}_{e}=dH_{1}H_{3}/(d{a_{3}a_{4}H}_{1}+{\mu a}_{3}^{2}a_{4}^{2})$

# Fitting results of model parameters

We used Metropolis-Hastings MCMC algorithm to estimate the model parameters. The fitting results are shown in Table S1 and Figure S1.

**Table S1.** Posterior means and posterior standard deviation (Std) of the estimated parameters.

| Parameter | Definition | Mean | Std |
| --- | --- | --- | --- |
| β | Human-to-human transmission rate | 0.115 | 0.034 |
| ψ | Ratio coefficient between environmental capacity of larval  mosquitoes and human population | 1.956 | 0.578 |
| *p* | Transmission rate from infected person fecal to larval | 0.231 | 0.002 |
| *Jv0* | Initial value of infected larval mosquitoes | 104 | 0.103 |
| *Ev0* | Initial value of the adult mosquito in exposed state | 165 | 0.115 |
| *Iv0* | Initial value of the adult mosquito in infection state | 88 | 0.091 |


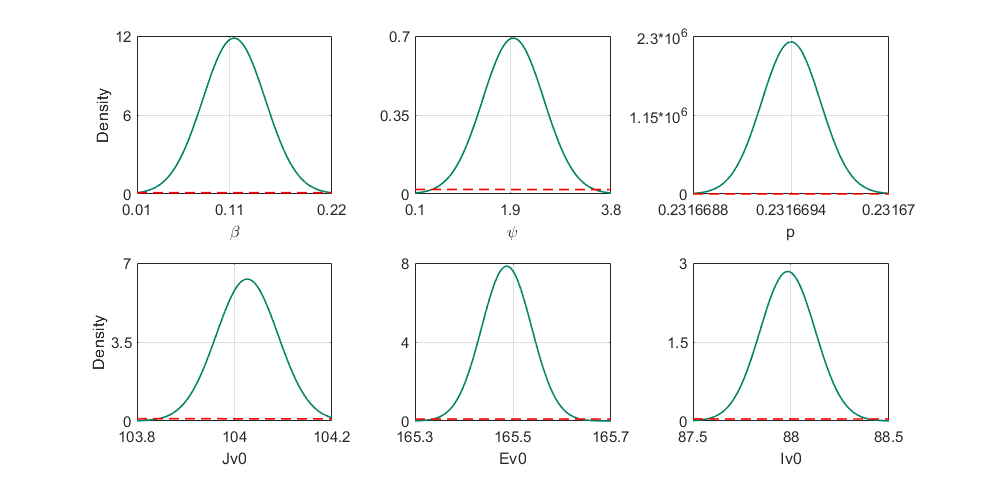


**Figure S1.** The prior and posterior distributions of the model parameters. The blue solid lines and red dotted lines represent the posterior and prior distributions, respectively.

# Uncertainty analysis

We perform uncertainty analysis to quantify the dependence of the estimated parameters on the output variables, and to characterize the response of outputs to parameters. Using the posterior distributions of the estimated parameters, we implemented the model for 1000 times and plotted the outputs as shown in Figure S2.

**
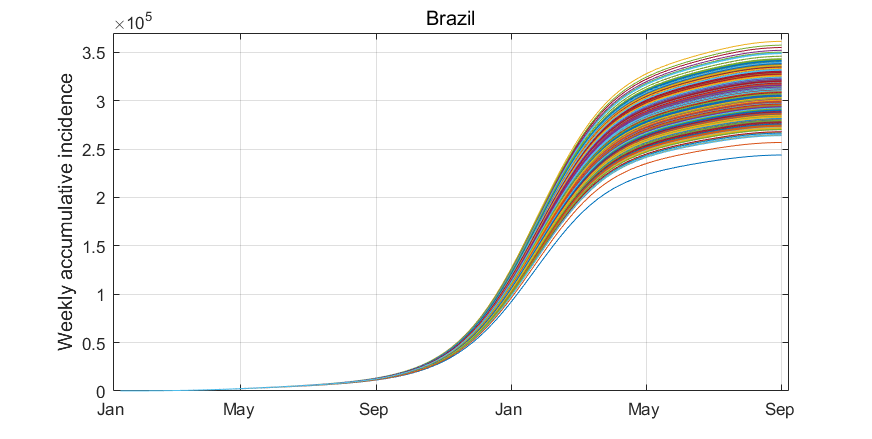
**

**Figure S2.** The output variables (weekly numbers of accumulate infected humans) by implementing the proposed model (1000 runs).

# Weekly report cases in Brazil

| 15 | 11 | 14 | 16 | 28 | 17 | 20 | 10 | 15 | 22 | 52 |
| --- | --- | --- | --- | --- | --- | --- | --- | --- | --- | --- |
| 113 | 152 | 170 | 313 | 450 | 417 | 739 | 853 | 1105 | 1104 | 1233 |
| 1231 | 1378 | 1335 | 1691 | 2521 | 3003 | 2592 | 2088 | 1740 | 1293 | 1048 |
| 829 | 614 | 604 | 569 | 512 | 525 | 468 | 465 | 489 | 576 | 689 |
| 861 | 1306 | 2256 | 4137 | 4805 | 4245 | 3575 | 1538 | 8161 | 8259 | 9447 |
| 10319 | 12717 | 14902 | 20980 | 20567 | 19743 | 17101 | 15192 | 12412 | 12164 | 10770 |
| 9512 | 7954 | 6980 | 5845 | 4728 | 3875 | 2781 | 2713 | 2316 | 1712 | 1408 |
| 1177 | 1055 | 899 | 826 | 671 | 637 | 610 | 509 | 417 | 390 | 341 |
| 318 | 236 | 211 | 224 | 211 | 202 | 146 | 90 | 25 | 5 | 0 |
| 0 | 0 | 0 | 0 | 0 |  |  |  |  |  |  |

**Table S2.** Weekly report cases in Brazil from 2015 to 2016
